# Realistic Silver Optical Constants for Plasmonics

**DOI:** 10.1038/srep30605

**Published:** 2016-07-29

**Authors:** Yajie Jiang, Supriya Pillai, Martin A. Green

**Affiliations:** 1 Australian Centre for Advanced Photovoltaics, School of Photovoltaic and Renewable Energy Engineering, University of New South Wales, Sydney, 2052, Australia

## Abstract

Silver remains the preferred conductor for optical and near-infrared plasmonics. Many high-profile studies focus exclusively on performance simulation in such applications. Almost invariably, these use silver optical data either from Palik’s 1985 handbook or, more frequently, an earlier Johnson and Christy (J&C) tabulation. These data are inconsistent, making it difficult to ascertain the reliability of the simulations. The inconsistency stems from challenges in measuring representative properties of pristine silver, due to tarnishing on air exposure. We demonstrate techniques, including use of silicon-nitride membranes, to access the full capabilities of multiple-angle, spectrometric-ellipsometry to generate an improved data set, representative of overlayer-protected, freshly-deposited silver films on silicon-nitride and glass.

Silver is predominantly the preferred material for applications in extraordinary optical transmission^1^,^2^,^3^, near-field and coherent microscopy^4^,^5^,^6^,^7^, far-field superlensing^8^,^9^, single photon emission^10^ and photovoltaic absorption-enhancement^2^,^11^,^12^,^13^. Almost invariably, simulations use Ag optical data from Palik’s 1985 handbook^14^ or, more commonly, an earlier Johnson and Christy (J&C) tabulation^15^. These data are inconsistent, giving vastly different results as previously noted^16^,^17^. J&C data give best results when low metal absorption is desired, while Palik’s best demonstrate strong absorption, with both sometimes used to emphasise different features in one paper^2^,^18^. The predominant use of two different, inconsistent Ag optical data sets presents a situation clearly needing resolution.

We have repeated the J&C experiments, obtaining similar reflection/transmission results, but measure obvious surface layer effects^17^. The original experiments were similarly corrupted by air exposure^15^,^16^, compounded by failure of the planned measurement approach at key wavelengths (additionally introducing 40% measurement uncertainty^15^), making continued use of J&C data difficult to justify. Lacking a strong challenge to their validity, their popularity is readily understood by comparing figures of merit^19^ for localised surface plasmon (LSP) and surface plasmon polariton (SPP) applications (Fig. 1a,b). J&C values are 6 times higher than Palik’s at near-infrared wavelengths. Palik’s data^14^ are likewise problematic, compiled from different samples including air-exposed, thin-film and bulk^16^,^17^, with readily-apparent inconsistencies (Fig. 1b).

Following thorough investigation of the physical, crystallographic and optical properties of Ag films, including comparison with single-crystal samples^17^,^20^, we generated an improved dataset representative of overlayer-protected, freshly deposited and annealed Ag films on silicon-nitride and glass, applying the full capabilities of modern multiple-angle spectroscopic ellipsometry (Fig. 1c,d; Supplementary Information Table). Prior to selection of the conditions for Ag deposition in this work, detailed studies were undertaken on the effect of deposition and annealing conditions and the resulting variable grain size upon the properties of the Ag films using electrical, optical, chemical and crystallographic characterisation. For films deposited onto nitride, a short pre-treatment of the nitride (400 °C, 15 mins) was found optimal for avoiding void formation in the Ag film, attributed to H evolution^20^. After deposition, a short low-temperature anneal (200 °C, 10 minutes) was found optimal, increasing grain size and reducing twin density (Fig. 2). More aggressive annealing created interfacial voids. A weak dependence of optical properties on grain size was observed (reported results correspond to conditions giving highest scattering times^17^,^20^). Single-crystal measurements gave marginally lower scattering times than optimised films attributed to remnant surface polishing damage, despite efforts to minimise^20^ (see Supplementary Information for details).

## Method

**Figure 3:**
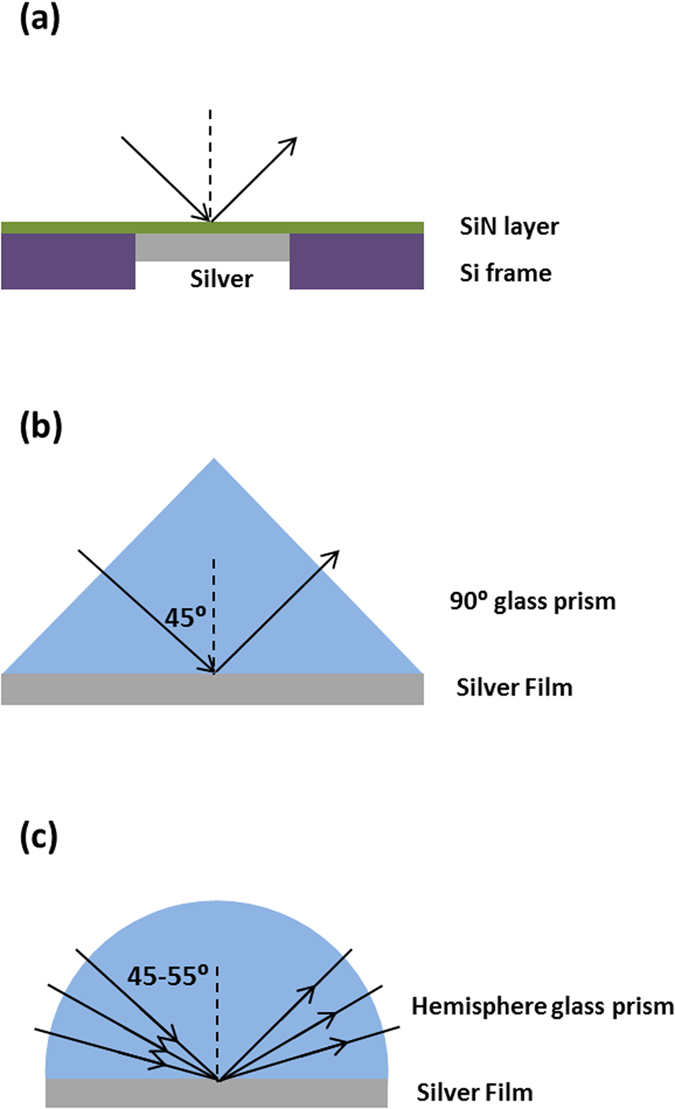
Samples used to produce a dominant coherent reflection for ellipsometry. (**a**) silicon nitride membrane supported by a silicon frame, with Ag deposited onto the unsupported membrane area. (**b**) right-angled fused silica prism. (**c**) hemispherical borosilicate glass prism (figures not to scale).

**Figure 4:**
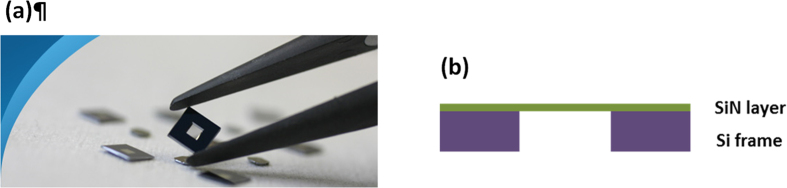
(**a**) SiNx membrane used as ‘substrate’ in the experiment. (**b**) A cross-sectional schematic of the SiNx membrane (not to scale).

**Figure 5:**
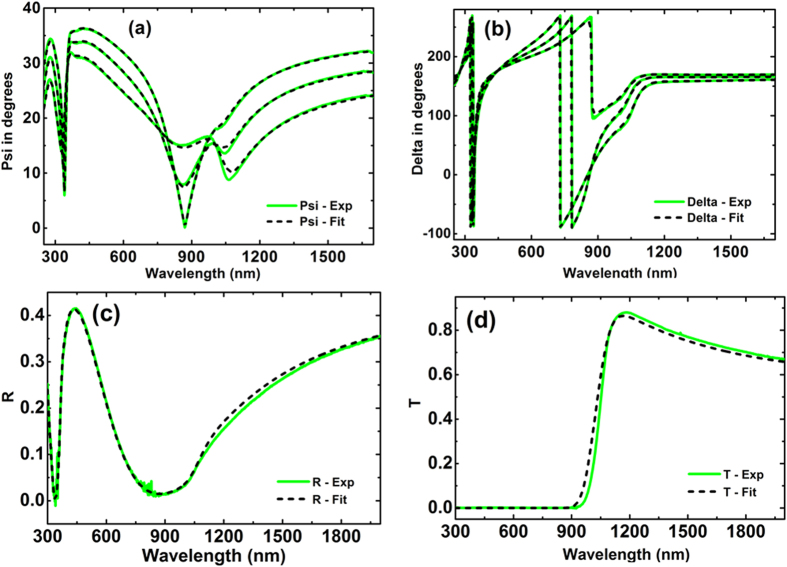
Comparison of experimental (green solid lines) and generated (black dashed lines) data of SiNx/Si/SiNx sample (**a**) amplitude component Ψ and (**b**) phase difference Δ. The ellipsometry data are collected from three incident angles 45°, 50° and 55°.

**Figure 6:**
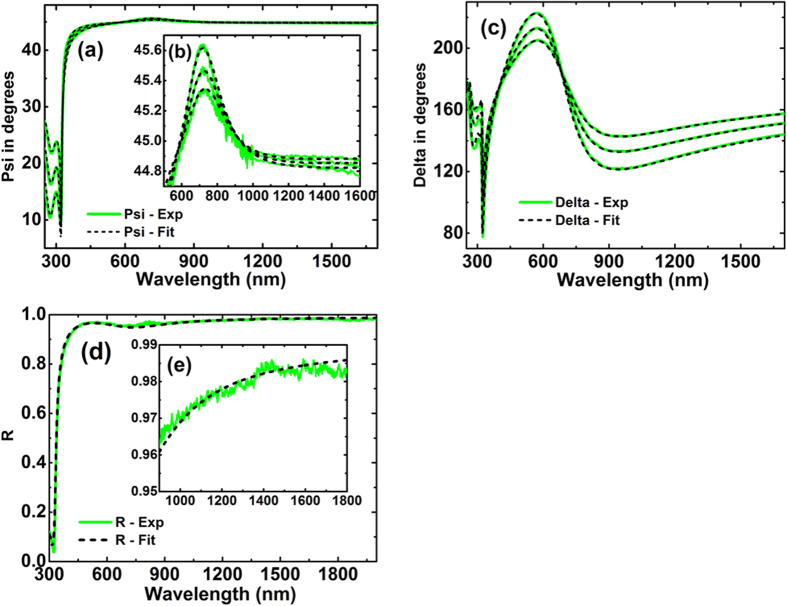
Comparison of experimental (green solid lines) and generated (black dashed lines) data of SiNx/Ag. (**a,b**) Amplitude component Ψ, (**c**) Phase difference Δ and (**d,e**) are the reflectance R. (**b,e**) are the zoomed insets of figs. (**a,d**) respectively. The ellipsometry data are collected from three incident angles 45°, 50° and 55°.

Our preferred measurement technique involved depositing films onto thin (75 nm) silicon-nitride membranes supported by silicon frames (Figs 3a and 4) producing coherent reflection from the protected Ag interface, enabling accurate ellipsometric analysis. Complementing data from three incidence angles, using two ellipsometers to cover overlapping wavelength ranges, near perpendicular reflectance was also measured. Using a Drude-Lorentz-Gaussian model^21^, experimental Psi, Delta (related to the differential change in polarisation amplitude and phase on reflection, respectively), and perpendicular *R* data could be fitted simultaneously over a wide range of wavelengths to a high degree of accuracy (Figs 5 and 6).

**Figure 7:**
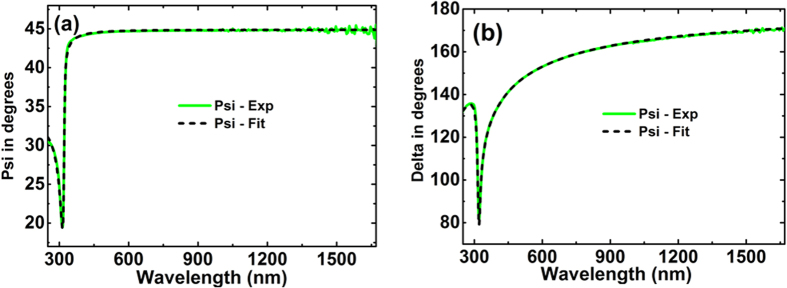
Comparison of experimental (green solid lines) and generated (black dashed lines) data of right angle glass prism/Ag for a 45° incident angle (**a**) Amplitude component Ψ and (**b**) Phase difference Δ.

**Figure 8:**
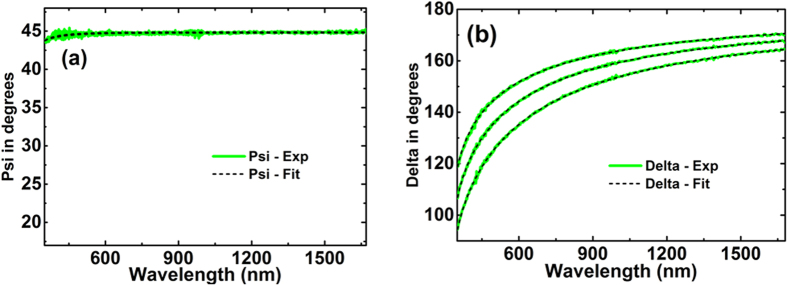
Comparison of experimental (green solid lines) and generated (black dashed lines) data of hemisphere glass prism/Ag corresponding to three incident angles of 45°, 50° and 55° (**a**) Amplitude component Ψ and (**b**) Phase difference Δ.

**Figure 9:**
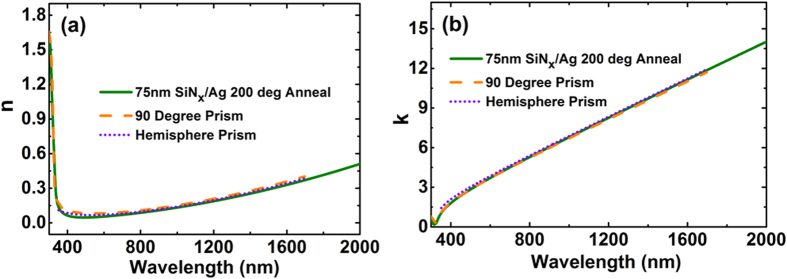
Comparison of results obtained by the three approaches used in this work. (**a**) Real part of the Ag refractive index, n. (**b**) Imaginary part of the Ag index, k.

Additional checks on these data and also on possible effects of different interfaces on Ag optical constants were investigated using two additional techniques that allowed accurate ellipsometric measurements, including effects of different overlayers. One used fused-silica right-angled prisms, restricting reflected light essentially to a coherent beam for 45° incidence; the other, hemispherical borosilicate prisms, allowing variable incidence angle (Figs 3b,c, 7 and 8). Extracted *n* and *k* values (Fig. 9) are essentially identical to membrane results, validating these and demonstrating no strong overlayer dependence.

For ellipsometry measurements, two J.A. Woollam M-2000 Ellipsometers covering the wavelength range of 210–1000 nm and 370–1690 nm respectively were used. Ellipsomety data collected from three angles of incidence (45°, 50° and 55°) and near perpendicular reflectance were additionally measured. Reflectance measurement was conducted using a Cary 500 spectrophotometer with a calibrated specular reference standard STAN-SSH-NIST (Ocean Optics). The fitting process was accomplished using WVASE^®^ which is a spectroscopic ellipsometry software from J. A. Woollam Co. that allows building a model based on the sample structure and describing the property of target materials using different oscillators^21^.

In the membrane approach, 75 nm thick silicon nitride (SiN_*x*_) membrane (Norcada) was used as a ‘substrate’ for Ag deposition. A silicon frame of 10 mm × 10 mm outer dimensions supports the 5 × 5 mm membrane (Fig. 4). The membrane can be treated as a top-capping layer if illumination is from the membrane side. This geometry results in a single coherent reflection from the protected Ag surface for ellipsometric analysis. Accurate values of the optical properties of the SiN_x_ film were determined using a 200 μm silicon wafer with 100 nm SiN_*x*_ film deposited on both sides sourced from the supplier and annealed under the same conditions as the membrane. Before Ag deposition, the membrane sample was pre-annealed at 400 °C for 15 minutes, ensuring a uniform and void free interface after Ag deposition. A 1 μm thick layer of optically opaque silver was thermally evaporated onto the membrane in a vacuum of 10^−6^ Torr at the rate of 20 Å/s. The source material was 99.999% pure silver from ESPI metals. Ellipsometry and reflectance measurements were performed immediately after the deposition of silver film from the silicon nitride side to determine the optical constants. The optical constants as well as film thickness in this case were fitted to the ellipsometry data together with reflection data using W-VASE^21^, as shown in Fig. 6.

Similar procedures were used for the right-angled and hemispherical prism approaches. The optical constants of the bare prisms were first extracted from ellipsometry measurements prior to silver deposition and the extracted values were found to be in excellent agreement with data sheet values from the vendors. In both cases, a 1 μm thick silver layer was deposited on the base of the prisms and the optical constants of Ag were determined subsequently.

### Simulations and Fittings

A typical membrane used in this study is shown in Fig. 4. A 200 μm silicon wafer with 100 nm SiN_*x*_ film deposited on both sides was sourced from the supplier to extract the optical constants of SiN_*x*_ film. Since the optical constants of silicon are well accepted^22^, characterization work could be focused on the SiN_*x*_ layer. A Cody-Lorentz oscillator was adopted to describe the property of silicon nitride, with 2 nm native silicon dioxide layers included in the model (SiN_*x*_/SiO_2_/Si/SiO_2_/SiN_*x*_) to achieve the best fit (minimum mean squared error between generated results and experimental data). A very good fit of the ellipsometry and spectrophotometric data was achieved as in Fig. 5.

Ag was deposited after pre-annealing the SiN_*x*_ membrane. The optical constants as well as film thickness in this case were fitted to the ellipsometry data together with reflection data in W-VASE using a Drude-Lorentz-Gaussian model^21^. As can be seen from Fig. 6, a good fit for the ellipsometry data was achieved with around 0.5% error for the reflectance data (Fig. 6c), reasonable for such measurements.

The ellipsometry measurements and corresponding simulated data shows good agreement as shown in Fig. 7 for the right angled prism and in Fig. 8 for the hemispherical prism. The latter prism showed absorption in the ultraviolet region, not considered in the fit. Multiple incident angles can be measured in the latter case when light is incident at the centre of the base increasing the number of measurements for the best fit. The model assumed the fused silica prism to be a semi-infinite incident medium. The extracted n and k values from this method are in good agreement with the values deduced fixing the nitride membrane (Fig. 9), validating this approach as well as demonstrating no strong dependence of Ag optical properties on the dielectric on which it was deposited.

Our finding was that when samples were prepared carefully to eliminate voids and surface layers, reproducible values of the silver optical constants could be obtained. We attribute much of the divergence reported in earlier literature to such artefacts.

A tabulation of the UNSW values and an Excel file including these values is included as Supplementary Information. Given the experimental difficulties with the J&C data extraction approach at the wavelengths usually of most interest, there is no sound basis for continued use of the J&C Ag data set, particularly for theoretical simulations where experimentally unattainably high performance is often predicted. Conversely, the Palik handbook data is too conservative, with the N&S and wider bandwidth UNSW data sets giving more realistic values for the properties of experimental films.

## Additional Information

**How to cite this article**: Jiang, Y. *et al*. Realistic Silver Optical Constants for Plasmonics. *Sci. Rep.* **6**, 30605; doi: 10.1038/srep30605 (2016).

## Supplementary Material

Supplementary Information

Supplementary Dataset 1

## Figures and Tables

**Figure 1:**
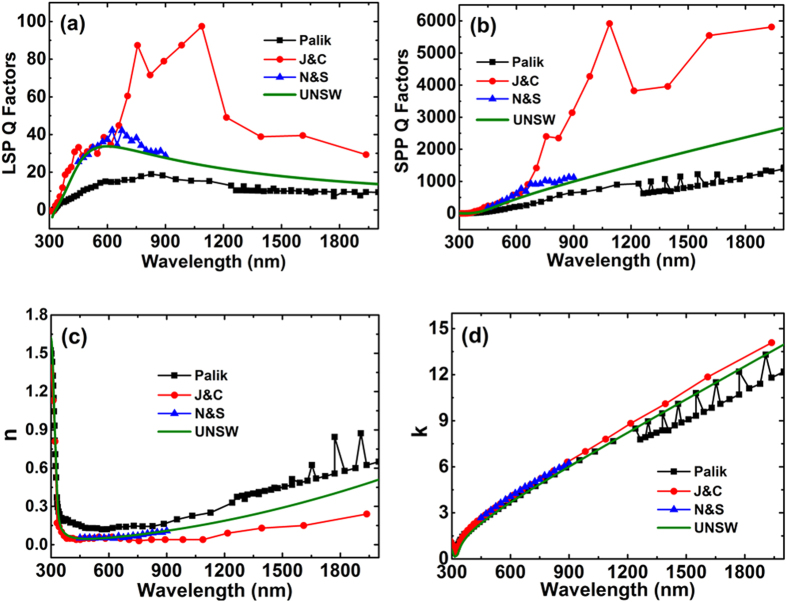
Ag plasmonic figures of merit and complex refractive index, showing large differences between the labelled data sets. (**a**) Localised surface plasmon (LSP) Q factor^19^ defined as |*(n*^*2*^* − k*^*2*^*)|/(2nk)* where *n* and *k* are real and imaginary parts of the Ag refractive index. (**b**) Surface plasmon polariton (SPP) Q factor^19^ defined as *(n*^*2*^ *− k*^*2*^)^2^*/(2nk)*. (**c**) Real part of Ag refractive index. (**d**) Imaginary part of Ag index. Shown are values for four labelled data sets, including the new “UNSW” dataset. This is in good agreement with that of Nash and Sambles (N&S)^16^, supporting these authors’ claim that theirs is the most accurate published to that date (1995). The high J&C figures of merit are thought due to experimental artefacts giving unrealistically low *n* values at near-infrared wavelengths.

**Figure 2:**
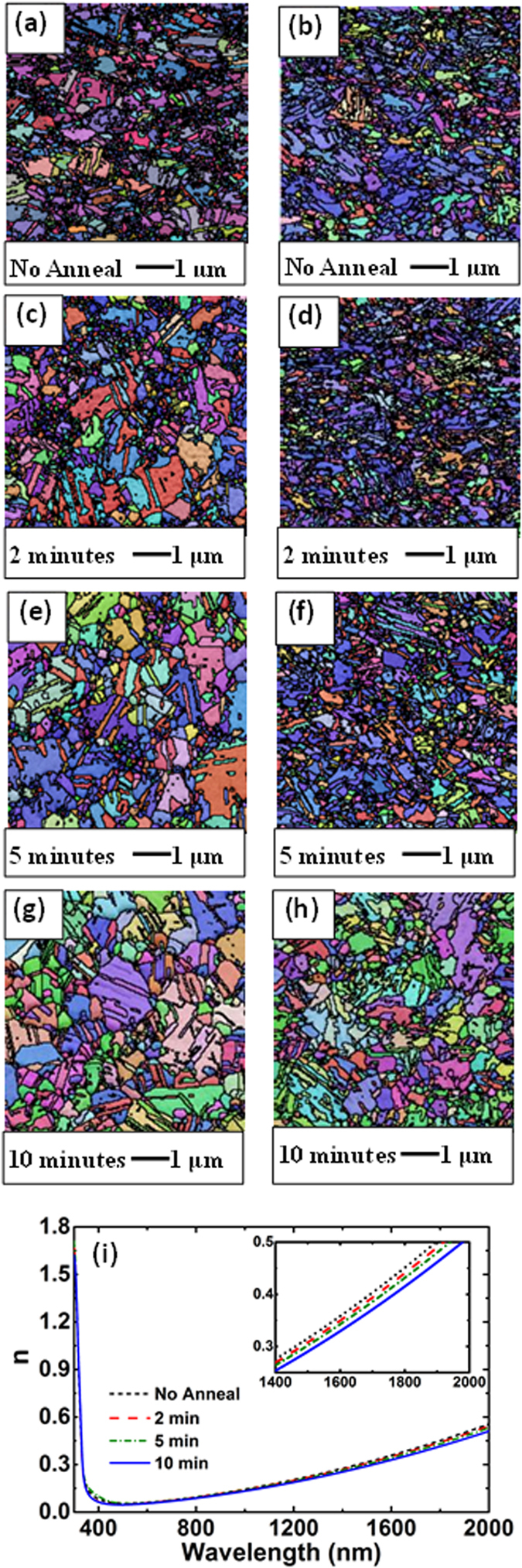
Grain boundary structure at free surface and at Ag/silicon-nitride interface after annealing and effect upon optical constants. (**a,c,e,g**) Electron back-scattering (ESBD) orientation maps at the Ag/air free surface as a function of annealing time at 200 ^°^C, with the different colours corresponding to different grain orientations. By drawing a set of line segments on the micrograph, the average grain intercept (AGI) distance was found to increase from 150 nm to 496 nm, as the annealing time increased from 0 to 10 minutes, with the AGI including twin boundaries increasing from 101 nm to 183 nm. (**b,d,f,h**) ESBD orientation maps at the Ag/silicon-nitride interface, with this area exposed by a modified template stripping approach^17^. AGI increased from 239 nm to 348 nm as annealing time increased from 0 to 10 minutes, with the AGI including twin boundaries increasing from 114 nm to 158 nm. Prolonged treatment at this temperature for up to 4 hours gave no further significant improvement. While the original as-deposited crystallographic quality at the Ag/nitride interface may have been similar or slightly better than that at the free surface, the free surface structure improved more significantly with annealing. (**i**) Effect of annealing upon the real part of the Ag refractive index, *n* (effect on imaginary part was negligible). Reducing *n* upon annealing corresponds to increased Q (Fig. 1) and also to increased scattering times. The AC scattering time^20^ increased from 13.5 fs to 14.5 fs on annealing, with the latter higher than the value of 14.2 fs measured in this work for a single crystal Ag sample using an additional approach.
