# Supplementary material for: Realistic Silver Optical Constants for Plasmonics
Source: Sci Rep. 2016 Jul 29;6:30605. doi: 10.1038/srep30605 (PMC4965786; doi:10.1038/srep30605)
Supplement: Supplementary Information [file srep30605-s1.doc]

**Supplementary Information**

**REALISTIC SILVER OPTICAL CONSTANTS FOR PLASMONICS**

Yajie Jiang, Supriya Pillai, Martin A. Green

*^1^Australian Centre for Advanced Photovoltaics, School of Photovoltaic and Renewable Energy Engineering, University of New South Wales, Sydney, Australia, 2052*

**Correspondence to m.green@unsw.edu.au*

**Single-Crystalline Measurements**

In addition to the annealed polycrystalline silver films discussed in the main text, we also measured a single-crystalline silver [(100) orientation] sample that was polished to give good surface quality with roughness of < 4nm as measured using Atomic Force Microscopy. The optical constants of single crystalline bulk silver were compared to data in Fig. S7. Fairly consistent results can be obtained between these two data sets. Slight variations are attributed to remnant surface polishing damage of single-crystal sample, despite efforts to minimise^1^.


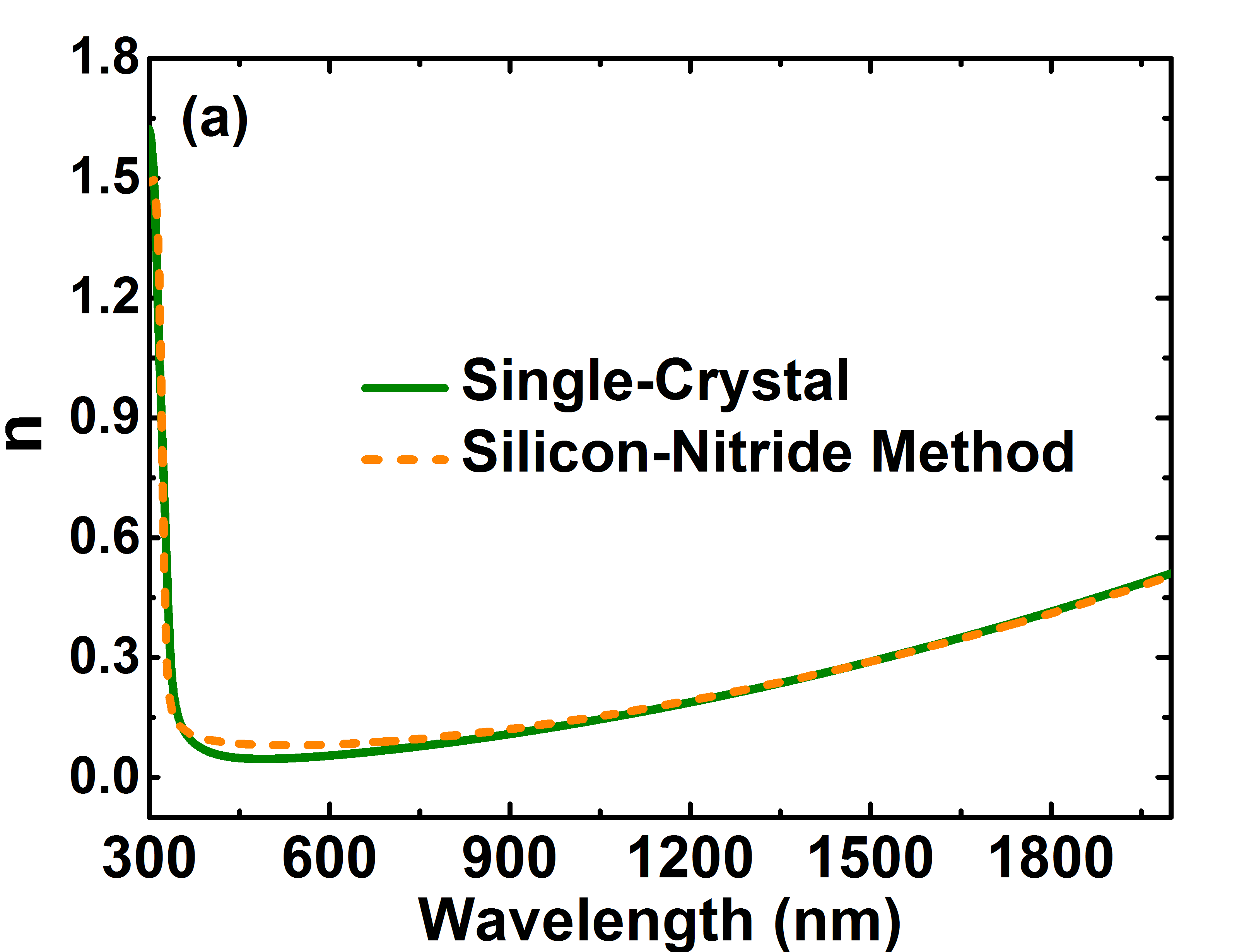

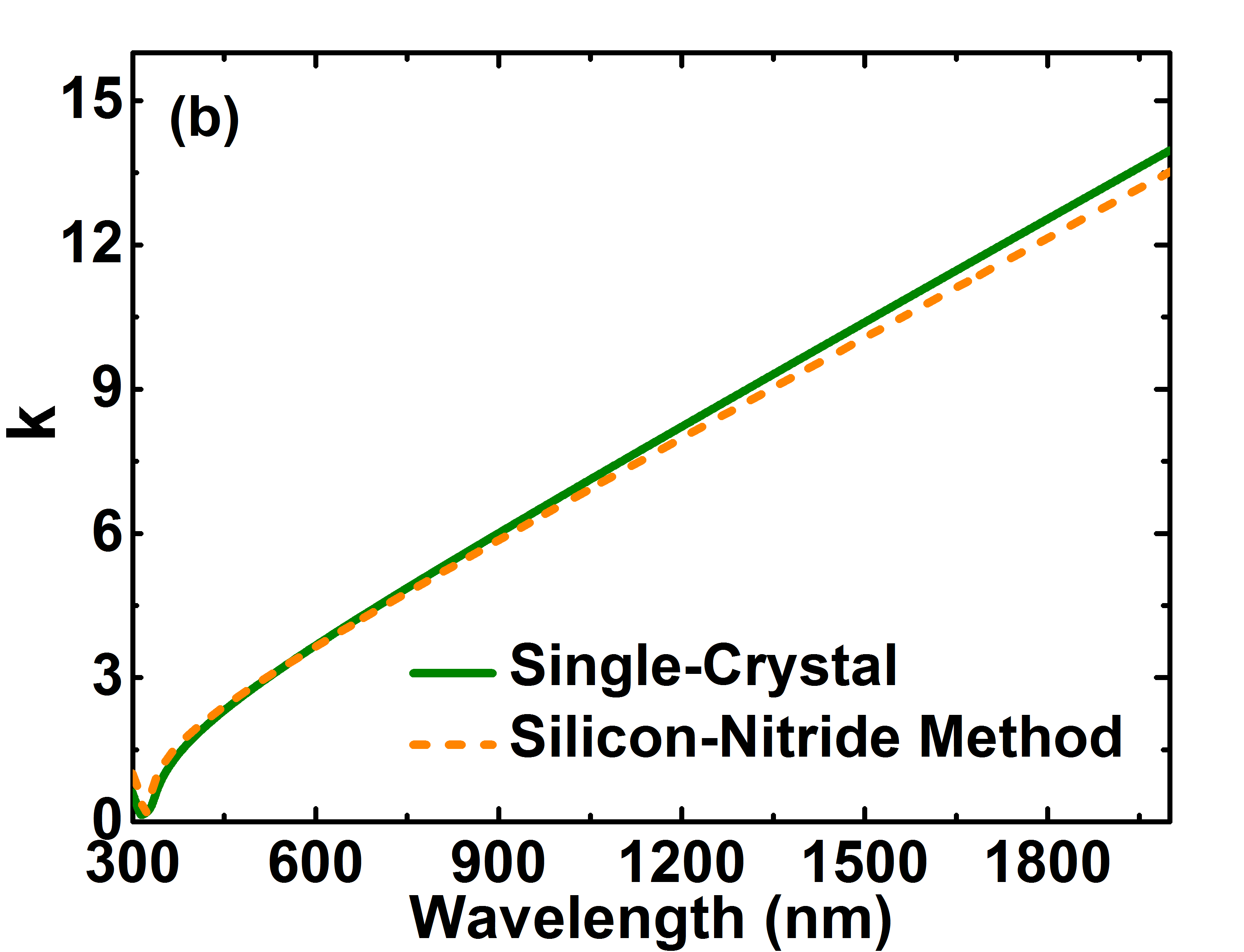


**Figure S6:** Comparison of results using single-crystal sample and annealed Ag films on silicon-nitride membrane described in main text. a, Real part of the Ag refractive index, *n*. b, Imaginary part of the Ag index, *k*.

An Excel file including tabulated values of the new UNSW values for Ag n and k is included

**References**

1. Jiang, Y. Optical properties for photovoltaics: silver and perovskite. PhD Thesis, University of New South Wales (2015).
